# Familial risk, abortion and their interactive effect on the risk of breast cancer--a combined analysis of six case-control studies.

**DOI:** 10.1038/bjc.1995.404

**Published:** 1995-09

**Authors:** N. Andrieu, S. W. Duffy, T. E. Rohan, M. G. Lê, E. Luporsi, M. Gerber, R. Renaud, D. G. Zaridze, Y. Lifanova, N. E. Day

**Affiliations:** Unité INSERM 351, Institut Gustave Roussy, Villejuif, France.

## Abstract

In a previous study in France, we reported that the relative risk of breast cancer associated with a family history of breast cancer was higher in those subjects with a history of abortions. The present study was undertaken to check the existence of this interaction in other studies and to investigate whether the interaction is modified by the time at which abortions occur. Data were obtained from six case-control studies in France, Australia and Russia, with information on family history of breast cancer and abortion for 2693 breast cancer cases and 3493 controls. The interaction effect was estimated in each study separately, then combined using a multivariate weighted average. The relative risk conferred by a family history of breast cancer increased with the number of abortions (1.8 for no abortion, 1.9 for one abortion, 2.8 for two or more). There was a significant interaction between total number of abortions and family history (P = 0.04), but this was no longer significant when adjusted for other risk factors. The familial risk was highest for those who had had an abortion before first childbirth (1.9 for abortion after first childbirth, 2.7 for abortion before first childbirth). The adjusted risk associated with family history was significantly higher in those with an abortion before first childbirth (P = 0.04). Our findings suggest a synergism between familial factors and abortion. The interaction was not substantially modified by the type of abortion (spontaneous or induced) but was modified by the time at which it occurred in relation to first childbirth. This suggests an effect of abortion itself rather than predisposition to abortion. Further studies of breast cancer cases, particularly among BRCA1 gene carriers and their families, could improve our understanding of this effect.


British Journal of Cancer (1995) 72, 744-751

© 1995 Stockton Press All rights reserved 0007-0920/95 $12.00

### Familial risk, abortion and their interactive effect on the risk of breast cancer – a combined analysis of six case-control studies

N Andrieu[1], SW Duffy[2], TE Rohan[3], MG Lê[1], E Luporsi[4], M Gerber[5], R Renaud[6], DG Zaridze[7], Y Lifanova[7] and NE Day[2]

[1]Unité INSERM 351, Institut Gustave Roussy, 39 rue Camille Desmoulins, 94805 Villejuif Cedex, France; [2]MRC Biostatistics Unit, Institute of Public Health, University Forvie Site, Robinson Way, Cambridge CB2 2SR, UK; [3]NCIC Epidemiology Unit, University of Toronto, 12 Queen's Park Crescent W, 3rd Floor, McMurrich Bldg, Toronto, Ontario, M5S 1A8, Canada; [4]Centre Alexis Vautrin, 54511 Vandoeuvre les Nancy Cedex, France; [5]Groupe d'Epidémiologie Métabolique, INSERM-CRLC, Epidaure, Rue des Apothicaires, Parc Euromédecine, 34094 Montpellier Cedex 5, France; [6]Département de Gynécologie Obstétricale, Hospices civils, 67000 Strasbourg, France; [7]Department of Epidemiology, Cancer Research Center, Russian Academy of Sciences, 24 Kashirskoje Shosse, 115478 Moscow, Russia.

**Summary** In a previous study in France, we reported that the relative risk of breast cancer associated with a family history of breast cancer was higher in those subjects with a history of abortions. The present study was undertaken to check the existence of this interaction in other studies and to investigate whether the interaction is modified by the time at which abortions occur. Data were obtained from six case-control studies in France, Australia and Russia, with information on family history of breast cancer and abortion for 2693 breast cancer cases and 3493 controls. The interaction effect was estimated in each study separately, then combined using a multivariate weighted average. The relative risk conferred by a family history of breast cancer increased with the number of abortions (1.8 for no abortion, 1.9 for one abortion, 2.8 for two or more). There was a significant interaction between total number of abortions and family history ($P = 0.04$), but this was no longer significant when adjusted for other risk factors. The familial risk was highest for those who had had an abortion before first childbirth (1.9 for abortion after first childbirth, 2.7 for abortion before first childbirth). The adjusted risk associated with family history was significantly higher in those with an abortion before first childbirth ($P = 0.04$). Our findings suggest a synergism between familial factors and abortion. The interaction was not substantially modified by the type of abortion (spontaneous or induced) but was modified by the time at which it occurred in relation to first childbirth. This suggests an effect of abortion itself rather than predisposition to abortion. Further studies of breast cancer cases, particularly among *BRCA1* gene carriers and their families, could improve our understanding of this effect.

**Keywords**: breast cancer; familial risk; induced abortion; spontaneous abortion

At present, there is no convincing evidence that abortion affects risk of breast cancer. Some studies have found a positive association between history of abortion and breast cancer, some a negative association and others no association (Kelsey and Horm-Ross, 1993). Results from most studies have been inconclusive, finding non-significant but suggestive associations. The difficulty in detecting the risk associated with abortion could be due to heterogeneity of the effect among the studied populations; in particular, familial factors may interact with history of abortion. Indeed, in a previous study, we reported that the risk of breast cancer associated with a family history of breast cancer increased in the presence of a history of abortion (Andrieu *et al.*, 1993). This interaction was statistically significant. Among women without a family history of breast cancer, no increased risk associated with abortion was observed, whereas among women with a family history of breast cancer, risk was increased 2-fold. The familial risk seemed to increase similarly for spontaneous and induced abortions. The interaction of family history of breast cancer and abortions or miscarriages has been examined only by two other studies (Parazzini *et al.*, 1992; Sellers *et al.*, 1993). Of these studies, one found an increased risk of breast cancer associated with spontaneous abortion among women with a family history of breast cancer (RR = 1.9) (Parazzini *et al.*, 1992), while the other found no association (Sellers *et al.*, 1993).

Although the reported significant interaction effects between familial factors and abortion may be a chance observation (Smith and Day, 1984), there is a plausible biological mechanism indicating that further investigation is worthwhile (Andrieu *et al.*, 1994). Specifically, it is of interest to check whether the interaction is present in studies from other environments, and to ensure that the sample size is sufficient to allow identification of the interaction (Smith and Day, 1984). We therefore decided to perform a combined analysis (using the raw data rather than published data) on six case-control studies, from various countries. The aim was to investigate the existence of the interaction and to investigate the effect of abortion before and after first full-term pregnancy through a study of modifications of the familial risk due to abortions.

Correspondence: N Andrieu, MRC Biostatistics Unit, Institute of Public Health, University Forvie Site, Robinson Way, Cambridge CB2 2SR, UK
Received 30 September 1994; revised 13 March 1995; accepted 10 April 1995.

### Materials and methods

The analysis included case-control studies from three countries, France, Australia and Russia. These data sets were chosen because they had information on family history of breast cancer and abortion. For all studies, family history of breast cancer was recalled by the subjects. Information on abortion history and on family history of breast cancer was not verified from medical records. The present analysis included 2693 breast cancer cases and 3493 controls. No family history in this exercise includes unknown family history. Most studies in the combined analysis have been published. The studies are briefly described in Table I, and the main design features are presented below.

### *Lê* et al. *(1984)*

This was a multicentre case-control study performed in France between 1981 and 1984 to investigate the relationship between oral contraceptive use and the risk of breast cancer.

Cases were between 20 and 45 years of age, with a histologically verified breast carcinoma diagnosed less than a year before the interview. Each case was matched with one control with respect to hospital, date of interview and age. This control was chosen from patients with non-malignant diseases, excluding benign breast disease and severe or moderate cervical dysplasia. Information was recorded on the occurrence of breast cancer in the family (sisters, mother, aunts and grandmothers) and the number of sisters and aunts.

*Richardson* et al. *(1991)*

This was a case-control study carried out in Montpellier (France) which focused on nutritional factors. Subjects were interviewed between 1983 and 1987. Cases were aged between 26 and 66 years old with histologically confirmed primary carcinoma of the breast who were hospitalised in the Montpellier Cancer Institute and had not previously undergone any therapy. Controls were women of the same age range admitted for the first time into three different wards: neurology and neurosurgery and general surgery. These women were attending for a first diagnosis and hence were not being currently treated for chronic diseases. Information was recorded on the occurrence of breast cancer in the family (sisters, mother and aunts) and the number of sisters and aunts.

*Clavel* et al. *(1991)*

The data were obtained from a case-control study in five French public hospitals between 1983 and 1987 to investigate the relationship between oral contraceptive use and the risk of breast cancer. Cases were between 20 and 56 years of age; they had a histologically confirmed infiltrating or *in situ* breast carcinoma. Three types of controls were eligible for each case: friends or colleagues, patients hospitalised for a non-malignant disease (except endocrinological diseases) and patients hospitalised for a malignant disease. The criteria for matching controls to cases were the centre, age at interview (± 5 years) and year of interview (± 14 months). Each case and her matching controls were interviewed by the same interviewer. The 111 controls with a malignant disease were excluded from the present analysis and the matching broken. Information was recorded on the occurrence of breast cancer in the family (sisters, mother, aunts and grandmothers) and the number of sisters and aunts.

*Luporsi (1988)*

This was a case-control study performed in Nancy Cancer Institute (France) between 1985 and 1987 to investigate the relationship between familial factors, alcohol, tobacco and obesity and the risk of breast cancer. Cases were between 24 and 83 years of age, with a histologically confirmed

**Table I** Studies included in the combined analysis

| *Study* | *Country* | *Number of cases* | *Number of controls* | *Age at interview* | *Time of interview* |
|---|---|---|---|---|---|
| Lê *et al.* (1984) | France | 265 | 265 | 22-46 | 1982-84 |
| Richardson *et al.* (1991) | France | 450 | 603 | 21-66 | 1983-87 |
| Clavel *et al.* (1991) | France | 495 | 785 | 20-56 | 1983-87 |
| Luporsi (1988) | France | 406 | 812 | 24-83 | 1985-87 |
| Rohan *et al.* (1988) | Australia | 451 | 451 | 20-74 | 1982-84 |
| DG Zaridze *et al.* (unpublished) | Russia | 626 | 577 | 23-82 | 1992-94 |
| Total | | 2693 | 3493 | | |

**Table II** Relative risk of breast cancer associated with the number of abortions

| *Study* | *Number of abortions* | *Cases* | *Controls* | *RR*[a] | *95% CI* |
|---|---|---|---|---|---|
| Lê *et al.* (1984) | 0 | 141 | 155 | 1 | |
| | 1 | 73 | 65 | 1.3 | 0.8-2.0 |
| | ⩾2 | 51 | 45 | 1.5 | 0.9-2.5 |
| Richardson *et al.* (1991) | 0 | 309 | 393 | 1 | |
| | 1 | 79 | 117 | 0.8 | 0.6-1.1 |
| | ⩾2 | 59 | 85 | 0.9 | 0.6-1.3 |
| | Unknown | 3 | 8 | – | – |
| Clavel *et al.* (1991) | 0 | 325 | 517 | 1 | |
| | 1 | 106 | 172 | 1.0 | 0.7-1.3 |
| | ⩾2 | 64 | 96 | 1.1 | 0.7-1.5 |
| Luporsi (1988) | 0 | 277 | 564 | 1 | |
| | 1 | 98 | 177 | 1.2 | 0.9-1.6 |
| | ⩾2 | 31 | 71 | 0.9 | 0.5-1.4 |
| Rohan *et al.* (1988) | 0 | 328 | 345 | 1 | |
| | 1 | 86 | 73 | 1.3 | 0.9-1.8 |
| | ⩾2 | 37 | 33 | 1.2 | 0.7-2.0 |
| DG Zaridze *et al.* (unpublished) | 0 | 139 | 102 | 1 | |
| | 1 | 112 | 101 | 0.8[b] | 0.6-1.2 |
| | ⩾2 | 374 | 372 | 0.7[b] | 0.5-1.0 |
| | Unknown | 1 | 2 | | |
| Combined data[c] | 0 | 1380 | 1974 | 1 | |
| | 1 | 442 | 604 | 1.1 | 0.9-1.2 |
| | ⩾2 | 242 | 330 | 1.1 | 0.9-1.3 |

[a]Adjusted for age at interview, age at menarche, age at first child, number of children, menopausal status and family history of breast cancer. [b]Crude odds ratios. [c]Combined analysis performed on the five sets of data for which variables for adjustment were available.

infiltrating breast carcinoma. Controls were women admitted into general surgery or general medicine wards. These women were examined to eliminate a diagnosis of cancer. Controls were matched to cases by age at interview (± 3 years), living area and occupational status. Each case was matched to two controls. Information was recorded on the occurrence of breast cancer in the family (sisters, mother, aunts and grandmothers) and the number of sisters and aunts.

*Rohan* et al. *(1988)*

This was a case–control study in South Australia. The cases were obtained from the population-based South Australian Central Cancer Registry between 1982 and 1984 to investigate the relationship between dietary intake and the risk of breast cancer. Cases were between 20 and 74 years of age, with a histologically verified first diagnosis of breast carcinoma. For each case, one control was selected at random from the electoral roll from among women of approximately the same age as that of the case at diagnosis. Study subjects were interviewed in their homes by trained interviewers. In addition to information on usual dietary intake, information on family history of cancer in sisters, mother and grandmothers was recorded. For the present study, information about first-degree relatives only was provided.

*DG Zaridze* et al. *(unpublished)*

The data were obtained from an ongoing case–control study being carried out in Moscow (Russia), which is focusing on diet, alcohol consumption and reproductive factors. Subjects were interviewed from 1992 to 1994. Cases were aged between 23 and 82 years old with histologically confirmed primary carcinoma of the breast and were recruited from four Moscow hospitals. Controls were women with minor non-chronic complaints registered in primary care polyclinics in Moscow. Information was recorded on the occurrence of breast cancer in the family (sisters, mother, aunts and grandmothers). Adjustment variables were not available for this analysis.

**Table III** Relative risk of breast cancer associated with abortion according to the nature of the abortion

| *Study* | *Number of abortions* | *Cases* | *Controls* | *RR*[a] | *95% CI* |
|---|---|---|---|---|---|
| Lê *et al.* (1984) | No abortion | 141 | 155 | 1 | |
| | Spontaneous | | | | |
| | 1 | 36 | 43 | 1.0 | 0.6–1.8 |
| | ⩾2 | 16 | 19 | 1.3 | 0.6–2.8 |
| | Induced | | | | |
| | 1 | 50 | 41 | 1.2 | 0.7–2.1 |
| | ⩾2 | 31 | 21 | 1.9 | 1.0–3.6 |
| Richardson *et al.* (1991) | | No information | | | |
| Clavel *et al.* (1991) | No abortion | 325 | 517 | 1 | |
| | Spontaneous | | | | |
| | 1 | 79 | 135 | 1.0 | 0.7–1.3 |
| | ⩾2 | 28 | 52 | 0.9 | 0.5–1.4 |
| | Induced | | | | |
| | 1 | 62 | 75 | 1.3 | 0.9–1.9 |
| | ⩾2 | 21 | 31 | 1.0 | 0.6–1.8 |
| Luporsi (1988) | No abortion | 277 | 564 | 1 | |
| | Spontaneous | | | | |
| | 1 | 83 | 164 | 1.0 | 0.8–1.4 |
| | ⩾2 | 27 | 62 | 0.9 | 0.5–1.4 |
| | Induced | | | | |
| | 1 | 21 | 26 | 1.8 | 1.0–3.5 |
| | ⩾2 | 5 | 7 | 1.9 | 0.5–6.9 |
| Rohan *et al.* (1988) | No abortion | 328 | 345 | 1 | |
| | Spontaneous | | | | |
| | 1 | 74 | 72 | 1.1 | 0.8–1.6 |
| | ⩾2 | 30 | 28 | 1.2 | 0.7–2.0 |
| | Induced | | | | |
| | 1 | 18 | 7 | 2.7 | 1.1–6.7 |
| | ⩾2 | 4 | 2 | 2.2 | 0.4–12.0 |
| DG Zaridze *et al.* (unpublished) | Spontaneous | | | | |
| | 0[b] | 521 | 490 | 1 | |
| | 1 | 87 | 72 | 1.1[c] | 0.8–1.6 |
| | ⩾2 | 18 | 14 | 1.2[c] | 0.6–2.4 |
| | Induced | | | | |
| | 0[d] | 162 | 125 | 1 | |
| | 1 | 123 | 96 | 1.0[c] | 0.7–1.4 |
| | ⩾2 | 340 | 354 | 0.7[c] | 0.6–1.0 |
| Combined data[e] | No abortion | 1071 | 1581 | 1 | |
| | Spontaneous | | | | |
| | 1 | 272 | 414 | 1.0 | 0.9–1.2 |
| | ⩾2 | 101 | 161 | 1.0 | 0.8–1.3 |
| | Induced | | | | |
| | 1 | 151 | 149 | 1.5 | 1.1–1.9 |
| | ⩾2 | 61 | 61 | 1.3 | 0.9–1.9 |

[a]Adjusted for age at interview, age at menarche, age at first child, number of children, menopausal status and family history of breast cancer. [b]Including induced only. [c]Crude odds ratios. [d]Including spontaneous only. [e]Combined analysis performed on the four sets of data for which variables for adjustment were available.

### Statistical methods

In the first stage of the analysis, each study was analysed separately using an unconditional or conditional logistic model according to the study design. Both crude and adjusted analyses, taking into account age at interview, age at menarche, number of children, age at first childbirth and menopausal status (plus family history when the main effects of abortion were studied), were performed. In the combined analysis, the relative risk estimates were combined by taking a multivariate weighted average. This method allows the point and interval estimates of relative risks to be obtained and provides tests of the effects on risk and tests of heterogeneity from study to study. Mathematical details are given elsewhere (Ewertz *et al.*, 1990).

For each study, in order to examine interactions of family history with abortion variables (number, type and time), the risk of a family history of breast cancer was calculated separately in each stratum of the abortion factor. In order to test the interaction, a chi-square homogeneity test was performed comparing the difference between the deviance of the above model and that of a model in which the familial risk was assumed the same in all strata. In the combined analysis, the interaction was tested as the statistical significance of the weighted average of the interaction terms (Breslow and Day, 1980; Ewertz *et al.*, 1990). Interactions with trends in quantitative variables were performed.

### Results

Firstly, main effects of abortion and family history were investigated. Table II shows the main effect of all abortions, Table III the effect according to the nature of the abortions and Table IV the effect according to the time of the first abortion in relation to the first childbirth. The main effect of abortion (Table II) was adjusted for age at interview, age at

**Table IV** Relative risk of breast cancer associated with abortion according to the time of the first abortion in relation to the first childbirth pregnancy

| *Study* | *Time of first abortion* | *Cases* | *Controls* | *RR*[a] | *95% CI* |
|---|---|---|---|---|---|
| Lê *et al.* (1984) | No abortion | 141 | 155 | 1 | |
| | After first full-term | 72 | 63 | 1.4 | 0.9–2.2 |
| | Before first full-term | 52 | 47 | 1.3 | 0.8–2.1 |
| Richardson *et al.* (1991) | No abortion | 311 | 396 | 1 | |
| | After first full-term | 93 | 127 | 0.9 | 0.7–1.3 |
| | Before first full-term | 36 | 61 | 0.7 | 0.4–1.1 |
| | Unknown | 10 | 19 | – | – |
| Clavel *et al.* (1991) | No abortion | 325 | 517 | 1 | |
| | After first full-term | 108 | 171 | 1.0 | 0.8–1.4 |
| | Before first full-term | 62 | 97 | 1.0 | 0.7–1.4 |
| Luporsi (1988) | No abortion | 277 | 564 | 1 | |
| | After first full-term | 92 | 193 | 1.1 | 0.8–1.4 |
| | Before first full-term | 37 | 55 | 1.2 | 0.6–2.2 |
| Rohan *et al.* (1988) | No abortion | 328 | 345 | 1 | |
| | After first full-term | 77 | 70 | 1.2 | 0.9–1.8 |
| | Before first full-term | 42 | 33 | 1.3 | 0.8–2.1 |
| | Unknown | 4 | 3 | – | – |
| DG Zaridze *et al.* (unpublished) | | No information | | | |
| Combined data | No abortion | 1382 | 1977 | 1 | |
| | After first full-term | 442 | 624 | 1.1 | 0.9–1.2 |
| | Before first full-term | 229 | 293 | 1.0 | 0.9–1.3 |
| | Unknown | 14 | 22 | – | – |

[a]Adjusted for age at interview, age at menarche, age at first child, number of children, menopausal status and family history of breast cancer.

**Table V** Main effect of family history of breast cancer on breast cancer risk

| *Study* | *Family history* | *Cases* | *Controls* | *RR*[a] | *95% CI* |
|---|---|---|---|---|---|
| Lê *et al.* (1984) | No | 203 | 227 | 1 | |
| | Yes[b] | 62 | 38 | 1.8 | 1.1–2.9 |
| Richardson *et al.* (1991) | No | 397 | 567 | 1 | |
| | Yes[c] | 50 | 28 | 2.8 | 1.7–4.5 |
| Clavel *et al.* (1991) | No | 396 | 674 | 1 | |
| | Yes[b] | 99 | 111 | 1.5 | 1.1–2.1 |
| Luporsi (1988) | No | 327 | 742 | 1 | |
| | Yes[b] | 79 | 70 | 2.8 | 1.9–4.1 |
| Rohan *et al.* (1988) | No | 410 | 424 | 1 | |
| | Yes[d] | 41 | 27 | 1.7 | 1.0–2.8 |
| DG Zaridze *et al.* (unpublished) | No | 558 | 554 | 1 | |
| | Yes[b] | 67 | 21 | 3.2[e] | 1.9–5.2 |
| Combined data | No | 2291 | 3188 | 1 | |
| | Yes | 398 | 295 | 1.9 | 1.6–2.3 |

[a]Adjusted for age at interview, age at menarche, age at first child, number of children, menopausal status. Family history of breast cancer is positive when at least one cancer occurred among: [b]sisters, mother, aunts and grandmothers; [c]sisters, mother and aunts; [d]sisters, mother. [e]Crude odds ratios.

menarche, age at first childbirth, number of children, menopausal status and family history of breast cancer (except for the Russian study, for which variables for adjustment were not available). In all studies there was no effect of abortion (induced and spontaneous abortion considered together). The combined analysis confirmed this observation with an odds ratio of 1.1 (95% CI 0.9–1.2) for one abortion and 1.1 (95% CI 0.9–1.3) for two or more abortions.

There was no effect of spontaneous abortion (Table III). Significant point estimates were observed in three out of five studies for induced abortions. The point estimates of relative risk varied from 0.7 (DG Zaridze *et al.*, unpublished) to 2.7 (Rohan *et al.*, 1988). The combined analysis showed an increased risk associated with experiencing one induced abortion, with an odds ratio of 1.5 (95% CI 1.1–1.9).

The relative risk of breast cancer associated with abortion was investigated according to the time of the first abortion in relation to first childbirth (Table IV). No difference in the risk of breast cancer was observed according to the time of first abortion, in individual studies or in the combined analysis.

The main effect of a family history of breast cancer is shown in Table V. The effect was significant in all studies. The odds ratio associated with a family history of breast cancer estimated from the combined analysis was 1.9 (95% CI 1.6–2.3).

The odds ratio associated with a family history of breast cancer increased as the number of abortions increased (Table VI) in five of the six studies. The combined analysis confirmed this, with an odds ratio associated with family history of 1.9 (95% CI 1.3–2.8) in those with one abortion and 2.8 (95% CI 1.7–4.7) in those with two or more abortions. The interaction was significant ($P = 0.04$) in a crude analysis but not when adjusted for age, age at menarche, age at first birth, number of children and menopausal status.

Similar results were obtained for spontaneous and induced abortions separately (Table VII). Table VIII shows the variation of the familial risk according to when the first abortion occurred in relation to the first childbirth. In four out of five studies, the familial risk was the highest when the first abortion occurred before the first childbirth. Within individual studies, the interaction was significant ($P = 0.03$) in the Australian study. This was confirmed in the combined analysis where the odds ratio was 1.9 (95% CI 1.3–2.8) when the first abortion had taken place after the first childbirth and 2.7 (95% CI 1.6–4.6) when the first abortion had taken place before the first childbirth. In the combined adjusted analysis there was a significant interaction between family history of breast cancer and abortion before first childbirth ($P = 0.04$).

In this study, we have chosen to present results as the effect of a family history stratified by the number, the nature or the time in relation to the first childbirth of the abortions. Indeed, we were interested by the modifications of the familial risk due to abortions. However, these results can be considered conversely as the effect of abortion stratified by family history and the results of the combined analyses are shown in Table IX.

### Discussion

This study found no effect on the risk of breast cancer of the total number of abortions, the number of spontaneous abortions or the time of abortion occurrence. However, in three studies and subsequently in the combined analysis, we found a significant increase in risk of breast cancer associated with induced abortion. These increases in risk were found in the two studies in which the proportion of subjects reporting induced abortion was the lowest (Luporsi, 1988, 4%; Rohan *et al.*, 1988, 2%), and in one study in which the proportion was average (Lê *et al.*, 1984, 23%). In the other studies, in which the proportion reporting induced abortion was 14% (Clavel *et al.*, 1991) and 78% (DG Zaridze *et al.*, unpublished), no increased risk was found. There is no obvious explanation for this discrepancy. A partial explanation is that induced abortion might be confounded with other risk factors in studies in which induced abortion is rare. The recent interview by Kelsey and Horm-Ross (1993) highlights the disparate results among studies concerning the association of abortions (spontaneous and induced) with breast cancer. Some studies have found a positive association, some a negative association and others have found no association

**Table VI** Relative risk of breast cancer associated with a family history of breast cancer by number of abortions

| *Study* | *Number of abortions* | *Without family history* Cases | *Without family history* Controls | *With family history* Cases | *With family history* Controls | *RR*[a] | *95% CI* | *P*[b] | *RR*[c] | *95% CI* | *P*[b] |
|---|---|---|---|---|---|---|---|---|---|---|---|
| Lê *et al.* (1984) | 0 | 106 | 129 | 35 | 26 | 1.7 | 1.0–3.2 | | 1.6 | 0.8–3.0 | |
| | 1 | 58 | 58 | 15 | 7 | 2.2 | 0.8–5.7 | NS | 2.1 | 0.7–5.9 | NS |
| | ⩾2 | 39 | 40 | 12 | 5 | 2.4 | 0.8–7.5 | | 2.1 | 0.7–6.9 | |
| Richardson *et al.* (1991) | 0 | 280 | 376 | 29 | 17 | 2.3 | 1.2–4.3 | | 2.5 | 1.3–4.8 | |
| | 1 | 67 | 110 | 12 | 7 | 2.8 | 1.1–7.5 | NS | 3.2 | 1.2–8.5 | NS |
| | ⩾2 | 50 | 81 | 9 | 4 | 3.7 | 1.1–12.5 | | 3.5 | 1.0–12.4 | |
| | Unknown | 3 | 7 | 0 | 1 | – | – | | – | – | |
| Clavel *et al.* (1991) | 0 | 266 | 445 | 59 | 72 | 1.4 | 0.9–2.0 | | 1.3 | 0.9–2.0 | |
| | 1 | 87 | 146 | 19 | 26 | 1.2 | 0.6–2.4 | NS | 1.2 | 0.6–2.3 | 0.08 |
| | ⩾2 | 43 | 83 | 21 | 13 | 3.1 | 1.4–6.8 | | 3.5 | 1.6–7.8 | |
| Luporsi (1988) | 0 | 222 | 516 | 55 | 48 | 2.8 | 1.8–4.4 | | 3.0 | 1.9–4.7 | |
| | 1 | 80 | 162 | 18 | 15 | 2.6 | 1.2–5.5 | NS | 2.6 | 1.2–5.6 | NS |
| | ⩾2 | 25 | 64 | 6 | 7 | 2.1 | 0.7–7.0 | | 2.1 | 0.6–7.0 | |
| Rohan *et al.* (1988) | 0 | 303 | 325 | 25 | 20 | 1.3 | 0.7–2.5 | | 1.4 | 0.8–2.7 | |
| | 1 | 76 | 69 | 10 | 4 | 2.3 | 0.7–7.6 | NS | 2.2 | 0.7–7.5 | NS |
| | ⩾2 | 31 | 30 | 6 | 3 | 1.9 | 0.4–8.5 | | 2.2 | 0.5–9.5 | |
| DG Zaridze *et al.* (unpublished) | 0 | 127 | 98 | 12 | 4 | 2.3 | 0.7–7.4 | | | | |
| | 1 | 102 | 98 | 10 | 3 | 3.2 | 0.9–11.9 | NS | | | |
| | ⩾2 | 329 | 358 | 45 | 14 | 3.5 | 1.9–6.5 | | | No information | |
| | Unknown | 0 | 2 | 1 | 0 | – | – | | | | |
| Combined data | 0 | 1304 | 1889 | 215 | 187 | 1.8 | 1.4–2.2 | | 1.8 | 1.4–2.2 | |
| | 1 | 470 | 643 | 84 | 62 | 2.0 | 1.4–2.9 | 0.04 | 1.9 | 1.3–2.8 | NS |
| | ⩾2 | 517 | 656 | 99 | 46 | 3.1 | 2.1–4.5 | | 2.8 | 1.7–4.7 | |

[a]Crude odds ratios. [b]Test for interaction between family history and number of abortions. [c]Adjusted for age at interview, age at menarche, age at first child, number of children and menopausal status.

**Table VII** Relative risk of breast cancer associated with a family history of breast cancer by the nature and number of abortions

| *Study* | *Number of abortions* | *Without family history* Cases | *Without family history* Controls | *With family history* Cases | *With family history* Controls | *RR*[a] | *95% CI* | *P*[b] | *RR*[c] | *95% CI* | *P*[b] |
|---|---|---|---|---|---|---|---|---|---|---|---|
| Lê *et al.* (1984) | No abortion | 106 | 129 | 35 | 26 | 1.8 | 1.0–3.3 | | 1.6 | 0.8–3.1 | |
| | Spontaneous | | | | | | | | | | |
| | 1 | 30 | 39 | 6 | 4 | 2.1 | 0.5–8.1 | NS | 2.9 | 0.7–12.0 | NS |
| | ⩾2 | 12 | 17 | 4 | 2 | 3.2 | 0.5–21.6 | | 2.5 | 0.4–17.8 | |
| | Induced | | | | | | | | | | |
| | 1 | 39 | 37 | 11 | 4 | 2.4 | 0.7–8.3 | NS | 1.9 | 0.5–7.1 | NS |
| | ⩾2 | 24 | 18 | 7 | 3 | 1.7 | 0.4–7.6 | | 1.4 | 0.3–7.0 | |
| Richardson *et al.* (1991) | | | | No data | | | | | | | |
| Clavel *et al.* (1991) | No abortion | 266 | 445 | 59 | 72 | 1.4 | 0.9–2.0 | | 1.4 | 0.9–2.0 | |
| | Spontaneous | | | | | | | | | | |
| | 1 | 59 | 116 | 20 | 19 | 2.1 | 1.0–4.2 | NS | 2.0 | 1.0–4.2 | NS |
| | ⩾2 | 21 | 3 | 7 | 7 | 2.1 | 0.7–6.9 | | 2.3 | 0.7–7.6 | |
| | Induced | | | | | | | | | | |
| | 1 | 51 | 63 | 11 | 12 | 1.1 | 0.5–2.8 | NS | 1.3 | 0.5–3.3 | NS |
| | ⩾2 | 12 | 27 | 9 | 4 | 5.1 | 1.3–19.7 | | 5.3 | 1.3–21.3 | |
| Luporsi (1988) | No abortion | 222 | 516 | 55 | 48 | 2.6 | 1.7–4.1 | | 2.7 | 1.7–4.3 | |
| | Spontaneous | | | | | | | | | | |
| | 1 | 69 | 148 | 14 | 16 | 2.1 | 0.9–4.6 | NS | 2.1 | 0.9–4.7 | NS |
| | ⩾2 | 21 | 56 | 6 | 6 | 2.5 | 0.7–8.6 | | 2.4 | 0.7–8.8 | |
| | Induced | | | | | | | | | | |
| | 1 | 16 | 25 | 5 | 1 } | 7.2 | 0.8–67.7 | NS | 5.0 | 0.5–50.6 | NS |
| | ⩾2 | 5 | 7 | 0 | 0 } | | | | | | |
| Rohan *et al.* (1988) | No abortion | 303 | 325 | 25 | 20 | 1.3 | 0.7–2.5 | | 1.4 | 0.8–2.6 | |
| | Spontaneous | | | | | | | | | | |
| | 1 | 65 | 68 | 9 | 4 | 2.4 | 0.7–7.6 | NS | 2.3 | 0.7–8.1 | NS |
| | ⩾2 | 24 | 25 | 6 | 3 | 2.1 | 0.5–9.3 | | 2.3 | 0.5–10.3 | |
| | Induced | | | | | | | | | | |
| | 1 | 21 | 9 | 1 | 0 | – | – | | – | – | |
| | ⩾2 | 0 | 0 | 0 | 0 | – | – | | – | – | |
| DG Zaridze *et al.* (1994) | Spontaneous | | | | | | | | | | |
| | 0[d] | 468 | 471 | 53 | 19 | 2.8 | 1.6–4.8 | | | | |
| | 1 | 73 | 72 | 14 | 0 } | 7.0 | 1.6–31.5 | NS | No information | | |
| | ⩾2 | 17 | 12 | 1 | 2 } | | | | | | |
| | Induced | | | | | | | | | | |
| | 0[e] | 147 | 119 | 15 | 6 | 2.0 | 0.8–5.4 | | | | |
| | 1 | 113 | 93 | 10 | 3 | 2.7 | 0.7–10.3 | NS | | | |
| | ⩾2 | 298 | 342 | 42 | 12 | 4.0 | 2.1–7.8 | | | | |
| Combined data | Spontaneous[f] | | | | | | | | | | |
| | 0 | 897 | 1415 | 174 | 166 | 1.7 | 1.4–2.2 | | 1.7 | 1.3–2.1 | |
| | 1 | 223 | 371 | 49 | 43 | 2.0 | 1.3–3.2 | NS | 2.0 | 1.3–3.1 | NS |
| | ⩾2 | 78 | 101 | 23 | 18 | 2.3 | 1.2–4.7 | | 2.4 | 1.2–4.9 | |
| | Induced[g] | | | | | | | | | | |
| | 0 | 372 | 574 | 94 | 98 | 1.5 | 1.1–2.0 | | 1.4 | 1.0–1.9 | |
| | 1 | 90 | 100 | 22 | 16 | 1.5 | 0.7–3.0 | NS | 1.6 | 0.8–3.4 | |
| | ⩾2 | 36 | 45 | 16 | 7 | 2.9 | 1.1–8.0 | | 3.1 | 1.1–8.5 | NS |

[a]Crude odds ratios. [b]Test for interaction between family history and number of spontaneous and induced abortions. [c]Adjusted for age at interview, age at menarche, age at first child, number of children, menopausal status. [d]Including induced only. [e]Including spontaneous only. [f]Performed on four studies: Lê *et al.* (1984), Clavel *et al.* (1991), Luporsi (1988), Rohan *et al.* (1988). [g]Performed on two studies: Lê *et al.* (1984), Clavel *et al.* (1991).

between abortion and breast cancer. One US study recently found that induced abortion could be involved in the aetiology of breast cancer (Daling *et al.*, 1994), although these results are still controversial (Rosenberg, 1994).

When the interaction was investigated, an increasing familial risk was found with increasing number of abortions in four out of five data sets. Similar results were obtained for spontaneous and induced abortion separately. When the familial risk was stratified by time of the first abortion in relation to first childbirth, a significantly increased familial risk was found when the first abortion was before the first childbirth. Most other interaction tests were not significant, suggesting the usual lack of power, even with large sample sizes, to detect interactions. It would have been interesting to look at the time of abortion relative to the time of first birth separately for spontaneous and induced abortions. Unfortunately, the number of cases was not large enough to perform such a double stratification in the interaction study.

In the crude combined analysis in which the familial risk was stratified by the number of abortions, the statistical significance of the interaction is not easily interpretable. Indeed, this analysis included the set of data (Clavel *et al.*, 1991) used in our previous study which generated the present study. We have performed combined analyses excluding the data set of Clavel *et al.* (1991). The statistical significance of the interaction of the familial relative risk with the number of abortions disappeared in the crude analysis. However, the point and interval estimates of familial relative risks still increased with the number of abortions increased (2.0 (1.6–2.7) for no abortion, 2.6 (1.7–4.0) for one abortion, 3.0 (2.0–4.7) for two or more abortions).

Because few women had experienced induced abortion in the studies of Rohan *et al.* (1988) and Luporsi (1988), the previous combined analysis, performed to estimate the familial relative risks according to the number of induced abortions, was done with only two data sets, those of Lé *et al.* (1984) and Clavel *et al.* (1991). Therefore when the data set of Clavel *et al.* (1991) is excluded, there is only one remaining.

The adjusted point and interval estimates of familial risks, according to the number of spontaneous abortions, and according to the time of first abortion relative to first birth

**Table VIII** Relative risk of breast cancer associated with a family history of breast cancer by time of first abortion in relation to the first full-term pregnancy

| Study | Time of first abortion | Without family history Cases | Without family history Controls | With family history Cases | With family history Controls | RR[a] | 95% CI | P[b] | RR[c] | 95% CI | P[b] |
|---|---|---|---|---|---|---|---|---|---|---|---|
| Lê *et al.* (1984) | No abortion | 106 | 129 | 35 | 26 | 1.7 | 1.0–3.2 | | 1.6 | 0.8–3.0 | |
| | After first full-term | 58 | 57 | 14 | 6 | 2.4 | 0.8–7.0 | NS | 2.3 | 0.7–7.3 | NS |
| | Before first full-term | 39 | 41 | 13 | 6 | 2.2 | 0.8–6.3 | | 2.1 | 0.7–6.2 | |
| Richardson *et al.* (1991) | No abortion | 280 | 376 | 29 | 17 | 2.4 | 1.3–4.4 | | 2.6 | 1.4–5.0 | |
| | After first full-term | 81 | 121 | 12 | 6 | 3.0 | 1.1–8.3 | NS | 3.4 | 1.2–9.7 | NS |
| | Before first full-term | 28 | 57 | 8 | 4 | 4.1 | 1.1–14.7 | | 3.9 | 1.1–14.7 | |
| | Unknown | 11 | 20 | 1 | 2 | – | – | | – | – | |
| Clavel *et al.* (1991) | No abortion | 266 | 445 | 59 | 72 | 1.4 | 0.9–2.0 | | 1.3 | 0.9–2.0 | |
| | After first full-term | 82 | 144 | 26 | 27 | 1.6 | 0.9–3.1 | NS | 1.7 | 0.9–3.2 | NS |
| | Before first full-term | 48 | 85 | 14 | 12 | 2.1 | 0.9–4.8 | | 2.2 | 1.0–5.1 | |
| Luporsi (1988) | No abortion | 222 | 516 | 55 | 48 | 2.8 | 1.8–4.3 | | 3.0 | 1.9–4.7 | |
| | After first full-term | 75 | 175 | 17 | 18 | 2.3 | 1.2–4.7 | NS | 2.3 | 1.1–4.7 | NS |
| | Before first full-term | 30 | 51 | 7 | 4 | 2.5 | 0.5–12.3 | | 2.9 | 0.6–15.0 | |
| Rohan *et al.* (1988) | No abortion | 303 | 325 | 25 | 20 | 1.3 | 0.7–2.5 | | 1.4 | 0.8–2.7 | |
| | After first full-term | 71 | 65 | 6 | 5 | 1.0 | 0.3–3.8 | 0.03 | 1.2 | 0.3–4.0 | 0.03 |
| | Before first full-term | 32 | 32 | 10 | 1 | 10.0 | 1.2–82.8 | | 10.6 | 1.3–88.0 | |
| | Unknown | 4 | 2 | 0 | 1 | – | – | | – | – | |
| DG Zaridze *et al.* (unpublished) | | | | No information | | | | | | | |
| Combined data | No abortion | 1177 | 1791 | 203 | 183 | 1.8 | 1.4–2.2 | | 1.8 | 1.4–2.2 | |
| | After first full-term | 367 | 562 | 75 | 62 | 2.0 | 1.4–2.8 | NS | 1.9 | 1.3–2.8 | 0.04 |
| | Before first full-term | 177 | 266 | 52 | 27 | 2.7 | 1.6–4.5 | | 2.7 | 1.6–4.6 | |
| | Unknown | 15 | 22 | 1 | 3 | – | – | | – | – | |

[a]Crude odds ratios. [b]Test for interaction between family history and abortion before first childbirth. [c]Adjusted for age at interview, age at menarche, age at first child, number of children and menopausal status.

**Table IX** Relative risk of breast cancer associated with the number, the nature and the time in relation to the first childbirth pregnancy of abortion according to the existence of a family history of breast cancer from the combined analyses

| | Without family history RR[b] | Without family history 95% CI | With family history RR[c] | With family history 95% CI | P[a] |
|---|---|---|---|---|---|
| Number of abortions | | | | | |
| 0 | 1 | – | 1 | – | |
| 1 | 1.1 | 0.9–1.2 | 1.1 | 0.7–1.7 | |
| ⩾2 | 1.0 | 0.8–1.2 | 1.6 | 0.9–2.6 | NS |
| Nature of the abortions | | | | | |
| No abortion | 1 | – | 1 | – | |
| Spontaneous | | | | | |
| 1 | 1.0 | 0.8–1.2 | 1.2 | 0.7–1.9 | |
| ⩾2 | 0.9 | 0.7–1.3 | 1.4 | 0.7–2.6 | NS |
| Induced | | | | | |
| 1 | 1.3 | 0.9–1.8 | 1.5 | 0.9–2.4 | |
| ⩾2 | 1.1 | 0.7–1.8 | 2.4 | 0.9–6.1 | NS |
| Time of the first abortion in relation to first childbirth | | | | | |
| No abortion | 1 | – | 1 | – | |
| After first full-term | 1.1 | 0.9–1.2 | 1.1 | 0.8–1.8 | |
| Before first full-term | 1.0 | 0.8–1.2 | 1.5 | 0.9–2.5 | 0.04 |

[a]Significance of interaction. [b]Adjusted for age at interview, age at menarche, age at first child, number of children, menopausal status.

were similar to those observed in the combined analyses including the data set of Clavel *et al.* (1991). These estimates are 1.9 (1.4–2.7) for no spontaneous abortion, 2.1 (1.1–3.8) for one spontaneous abortion, 2.5 (1.0–5.9) for two or more spontaneous abortions, 2.1 (1.6–2.7) for no abortion, 2.1 (1.3–3.4) for first abortion after first birth and 3.3 (1.6–6.5) for first abortion before first birth. Like the effect of total number of abortions, the statistical significance of the interaction with the time of the first abortion disappeared. The differences in the significance may therefore be due to the reduction in the number of cases.

Two studies were characterised by a younger age range because the aim of these studies was to determine the effect of oral contraceptive use on breast cancer risk in young women. The aim of the four others was to determine the association between diet and breast cancer. This difference in age range does not seem to be a problem in our study. Adjustment for age was performed in the adjusted analyses and the results seem to hold for both groups of studies.

The measurement of family history of breast cancer was not homogeneous from study to study. Four studies recorded information in first- and second-degree relatives (Lê *et al.*, 1984; Luporsi, 1988; Clavel *et al.*, 1991; DG Zaridze, unpublished). One study recorded information in first- and second-degree relatives but not in grandmothers (Richardson *et al.*, 1991), and one study in first-degree relatives (Rohan *et al.*, 1988). Therefore the risk estimated from combined analysis measured the familial risk of breast cancer without a precise definition of the familial relationship. The heterogeneity in the method of measuring family history might have induced errors in the interaction estimation if genetic susceptibility differs according to the type of familial relationship with an affected relative, and if the abortion effect differs according to the type of genetic susceptibility. The occurrence of both conditions is necessary for there to be errors in the estimation of the interaction term. Byrne *et al.* (1991) found that different factors could modify in different directions the effects of an affected mother and the effects of an affected sister. However, as abortions were not studied by Byrne *et al.* (1991), this sheds no light on the possible error. Thus, further studies could be performed in order to investigate the variation of the interaction according to the type of familial relationship of affected relative.

No family history in this exercise included unknown family history. This measurement of family history of breast cancer could bias the results if cases were more aware of such a history than controls. In several studies, however, cases and controls had a similar proportion of relatives with an unknown cancer status and this proportion is small (among first-degree relatives: Lê *et al.*, 1.5%; Richardson *et al.*, 3%;

Clavel et al., 2%; Luporsi, 3%). Moreover, Go et al. (1983), in a study which involved contacting relatives or reviewing records to verify reports, found no difference between the accuracy of reports from women who themselves had had breast cancer and those who had not. Also, although this bias might affect the estimation of the relative risk for the main effect of family history, there is no reason to assume that it would vary according to the number of abortions, the time of abortions or the type of abortions.

The corresponding bias (Lindefors-Harris et al., 1991) caused by cases being more aware of the abortions than controls might explain the increased risk of breast cancer found in some studies for induced abortions (Lê et al., 1984; Luporsi, 1988; Rohan et al., 1988) but, again, there is no reason to assume that this bias would vary according to the existence or not of a family history of breast cancer.

The interaction of a family history of breast cancer with abortions or miscarriages has been examined by two other independent studies (Parazzini et al., 1992; Sellers et al., 1993); one found an increased risk associated with spontaneous abortion only (Parazzini et al., 1992).

Using another study design, two studies have investigated the effect of abortions on breast cancer by comparing cases with blood-related controls. In the first study, we compared (Andrieu and Demenais, 1994) 160 cases with sister controls and showed that the relative risk associated with the number of abortions increased (spontaneous and induced). Moreover, the relative risk was 1.5 times higher than the one estimated by using unrelated controls. In the second study, Laing et al. (1994) analysed 138 pairs of cases/sister controls and showed an increase in breast cancer risk associated with both spontaneous and induced abortions. Although the amplitude of the two relative risks was similar, only the relative risk associated with induced abortion was significant. In that study, comparison with unrelated controls has also been done and no increased risk has been found. Thus, the relative risks with respect to abortion history differ according to the type of controls (blood-related control or unrelated control), suggesting an interaction between family history of breast cancer and history of abortion.

In our study, the risk associated with a family history increased for both spontaneous and induced abortions and especially when abortion occurred before the first childbirth. These findings suggest firstly an effect of abortion itself rather than predisposition to abortion and secondly an effect of the time when abortion occurs. Our previous hypothesis seems to fit well with the results. This hypothesis is that abortion may be a catalytic event which exacerbates an existing familial risk of breast cancer. Indeed, the first 3 months of a pregnancy (especially of the first pregnancy) is a period during which undifferentiated cells increase in the breast tissue. If, because of abortion, the first trimester is not followed by differentiation which should ensue during the second and the third trimesters, then there is an increase in the number of vulnerable cells. These cells are vulnerable because they are hypersensitive to genotoxic carcinogens (Krieger, 1989). If we suppose that breast cancer is the consequence of successive genetic mutations and that women with a family history of breast cancer carry one of these mutations in their germ line (Knudson, 1971), then an increase in the number of sensitive cells may be responsible for a strong increase in the risk of breast cancer. Thus, abortion might be associated with an increased risk of breast cancer, whatever the underlying, incompletely penetrant genetic susceptibility. The risk would be expected to vary according to the term of the terminated pregnancy, the time between abortion and a further full-term pregnancy and also the age at abortion. Consequently, to verify this, further studies of breast cancer cases, particularly among *BRCA1* gene carriers and their families, with detailed information on reproductive and familial factors, could improve our understanding of this effect.

**Acknowledgements**
We thank Dr AM Goldstein for helpful comments on the manuscript. This study was made possible by an MRC-INSERM exchange grant.

**References**

ANDRIEU N, CLAVEL F, AUQUIER A and 7 others. (1993). Variations in the risk of breast cancer associated with a family history of breast cancer according to age at onset and reproductive factors. *J. Clin. Epidemiol.*, **46**, 973-980.

ANDRIEU N, CLAVEL F, GAIRARD B and 5 others. (1994). Familial risk of breast cancer and abortion. *Cancer Detect. Prevent.*, **18**, 51-55.

ANDRIEU N AND DEMENAIS F. (1994). Role of genetic and reproductive factors in breast cancer. *Genet. Epidemiol.*, **11**, 285.

BRESLOW NE AND DAY NE. (1980). The analysis of case-control studies. In *Statistical Methods in Cancer Research*. Davis W. (ed.) pp. 192-200. IARC: Lyon.

BYRNE C, BRINTON LA, HAILE RW AND SCHAIRER C. (1991). Heterogeneity of the effect of family history on breast cancer risk. *Epidemiology*, **2**, 276-284.

CLAVEL F, ANDRIEU N, GAIRARD B and 7 others. (1991). Oral contraception and breast cancer: a French case-control study. *Int. J. Epidemiol.*, **20**, 32-38.

DALING JR, MALONE KE, VOIGT LF, WHITE E AND WEISS NS. (1994). Risk of breast cancer among young women relationship to induced abortion. *J. Natl Cancer Inst.*, **86**, 1584-1592.

EWERTZ M, DUFFY SW, ADAMI H-O and 6 others. (1990). Age at birth, parity and risk of breast cancer: a meta-analysis of 8 studies from the Nordic countries. *Int. J. Cancer*, **46**, 597-603.

GO RCP, KING MC, BAILEY-WILSON J, ELSTON RC AND LYNCH HT. (1983). Genetic epidemiology of breast and associated cancer in high risk families. I. Segregation analysis. *J. Natl Cancer Inst.*, **71**, 455-561.

KELSEY JL AND HORM-ROSS PL. (1993). Breast cancer: magnitude of the problem and descriptive epidemiology. *Epidemiol. Rev.*, **15**, 7-15.

KNUDSON AG. (1971). Mutation and cancer: statistical study of retinoblastomas. *Proc. Natl Acad. Sci. USA*, **68**, 820-823.

KRIEGER N. (1989). Exposure, susceptibility and breast cancer risk: a hypothesis regarding exogenous carcinogens, breast tissue development, and social gradients, including black/white differences, in breast cancer incidence. *Breast Cancer Res. Treat.*, **13**, 205-223.

LAING AE, BONNEY GR, ADAMS-CAMPBELL L and 6 others. (1994). Reproductive and lifestyle risk factors for breast cancer in African-American women. *Genet. Epidemiol.*, **11**, 300.

LÊ MG, BACHELOT A, DOYON F, KRAMAR A AND HILL C. (1984). Oral contraceptive use and breast cancer or cervical cancer: preliminary results of a French case-control study. In *Hormones and Sexual Factors in Human Cancer Aetiology*, Wolff J-P and Scott JD. (eds) pp. 139-147. Elsevier Science: Amsterdam.

LINDEFORS-HARRIS B-M, EKLUND G, ADAMI H-O AND MEIRIK O. (1991). Response bias in a case-control study: analysis utilizing comparative data concerning legal abortions from two independent Swedish studies. *Am. J. Epidemiol.*, **134**, 1003-1008.

LUPORSI E. (1988). Breast cancer and alcohol. PhD thesis, University of Paris-Sud.

PARAZZINI F, LA VECCHIA C, NEGRI E, FRANCESCHI S AND BOCCIOLONE L. (1992). Menstrual and reproductive factors and breast cancer in women with family history of the disease. *Int. J. Cancer*, **51**, 677-681.

RICHARDSON S, GERBER M AND CENEE S. (1991). The role of fat, animal protein and vitamin consumption in breast cancer. A case-control study in Southern France. *Int. J. Cancer*, **48**, 1-9.

ROHAN T, MCMICHAEL AJ AND BAGHURST PA. (1988). A population-based case-control study of diet and breast cancer in Australia. *Am. J. Epidemiol.*, **128**, 478-489.

ROSENBERG L. (1994). Induced abortion and breast cancer: more scientific data are needed. *J. Natl Cancer Inst.*, **86**, 1569-1570.

SELLERS TA, POTTER JD, SEVERSON RK and 4 others. (1993). Difficulty becoming pregnant and family history as interactive risk factors for postmenopausal breast cancer: the Iowa Women's Health Study. *Cancer Causes Control*, **4**, 21-28.

SMITH PG AND DAY NE. (1984). The design of case-control studies: the influence of confounding and interaction effects. *Int. J. Epidemiol.*, **13**, 356-365.


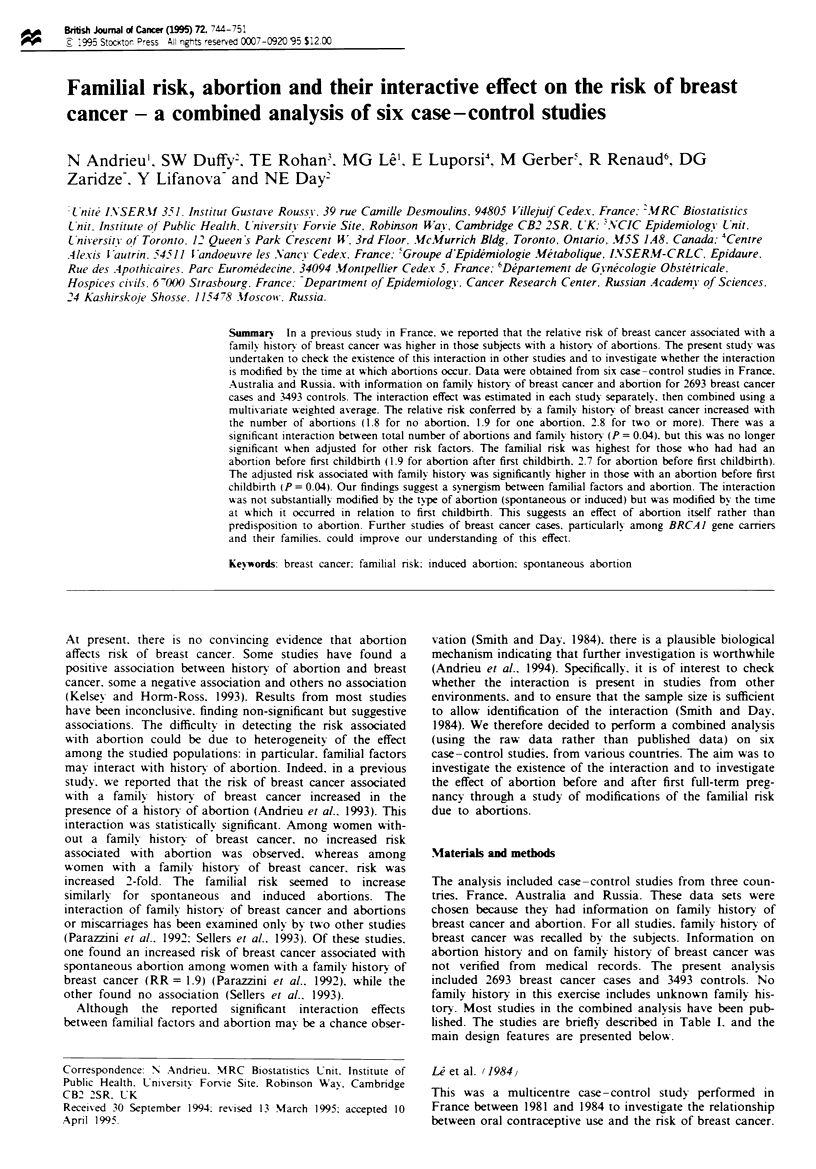

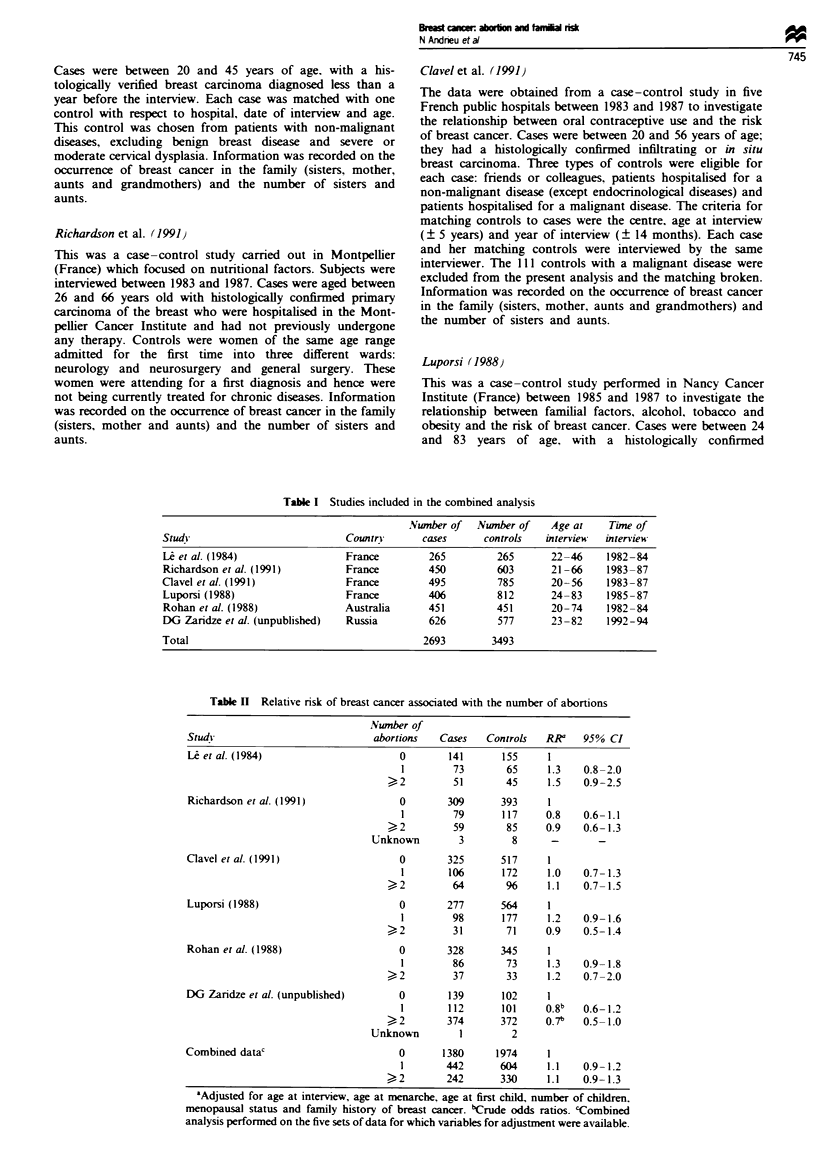

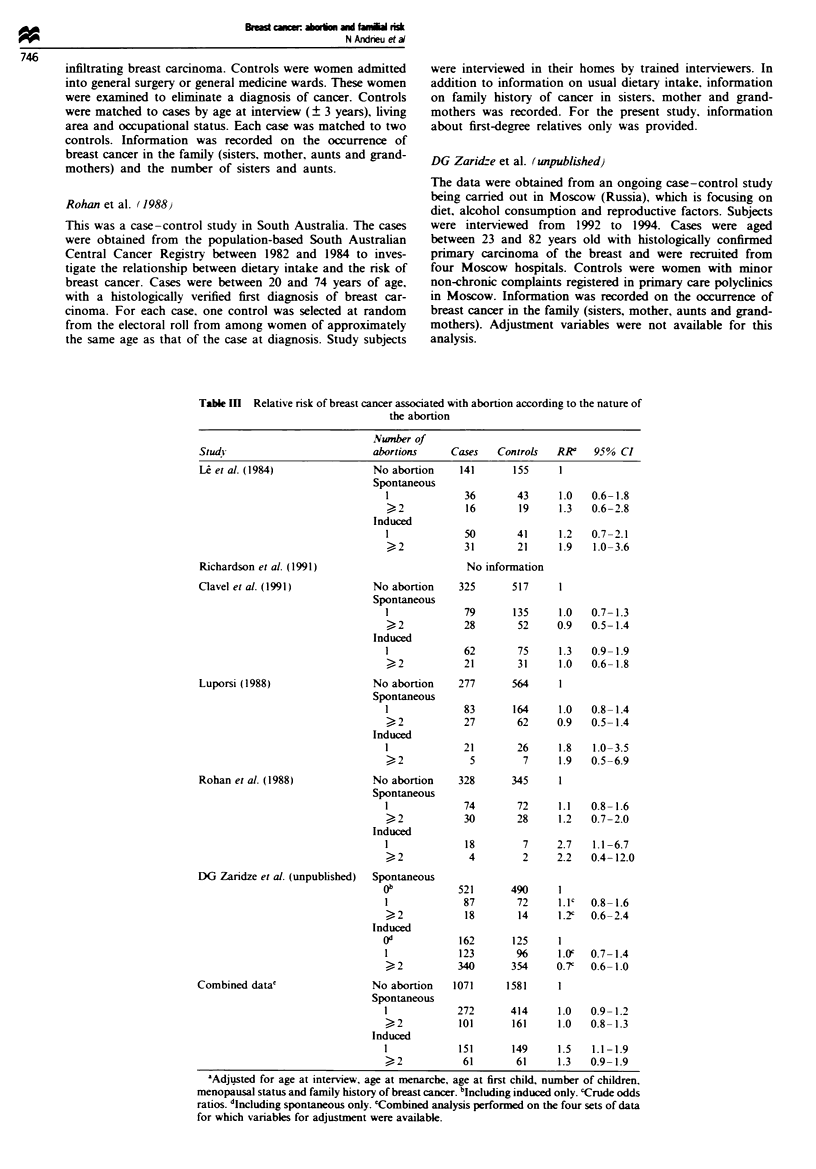

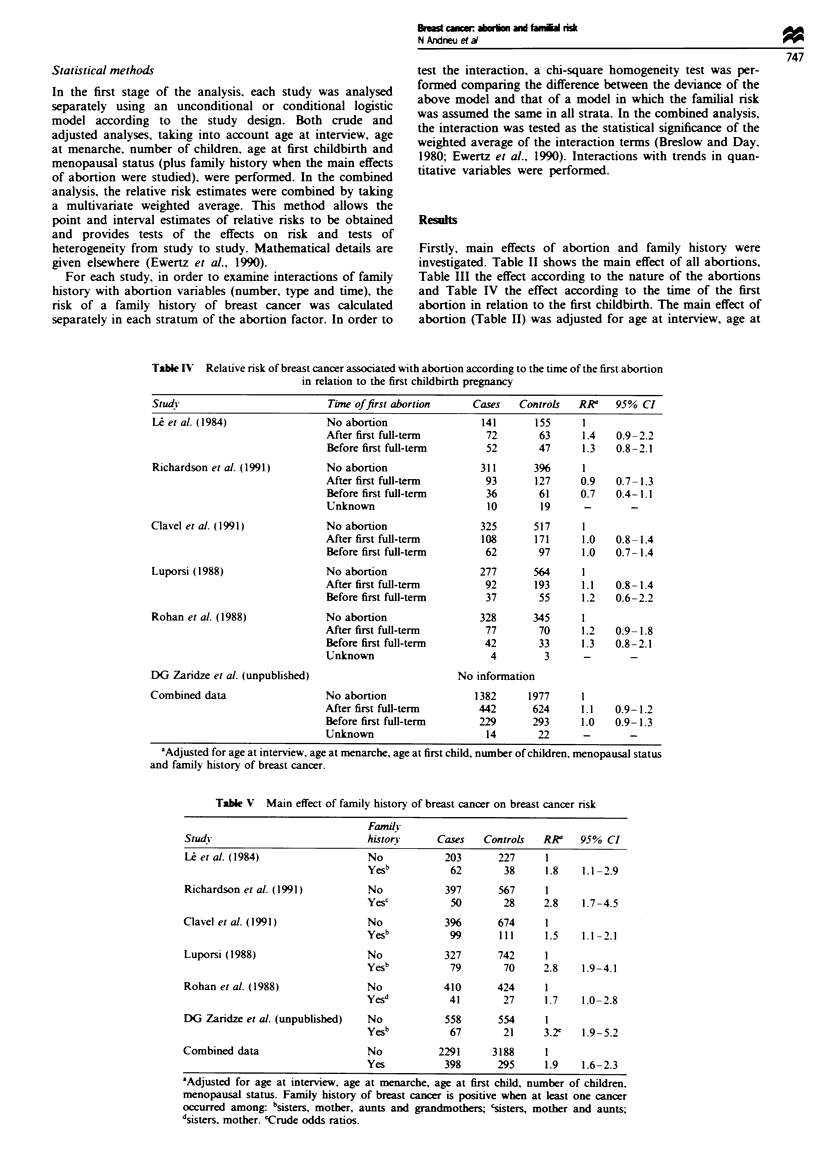

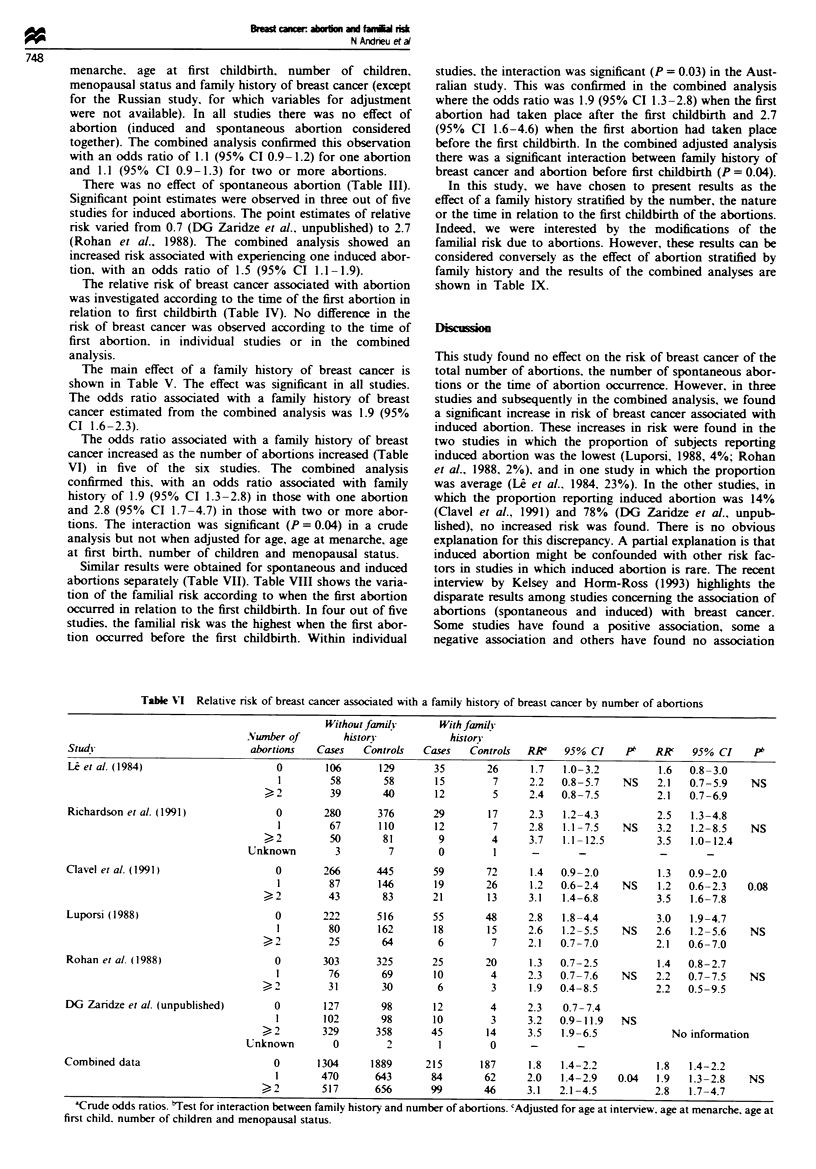

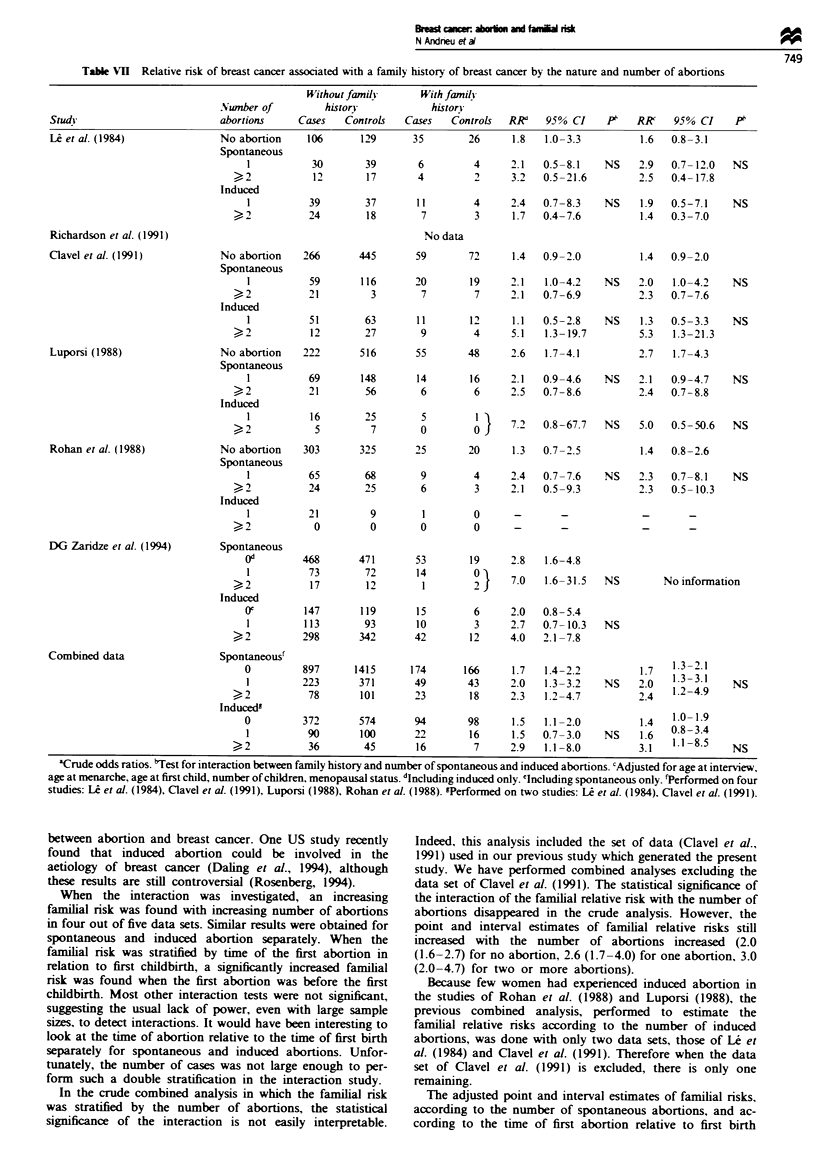

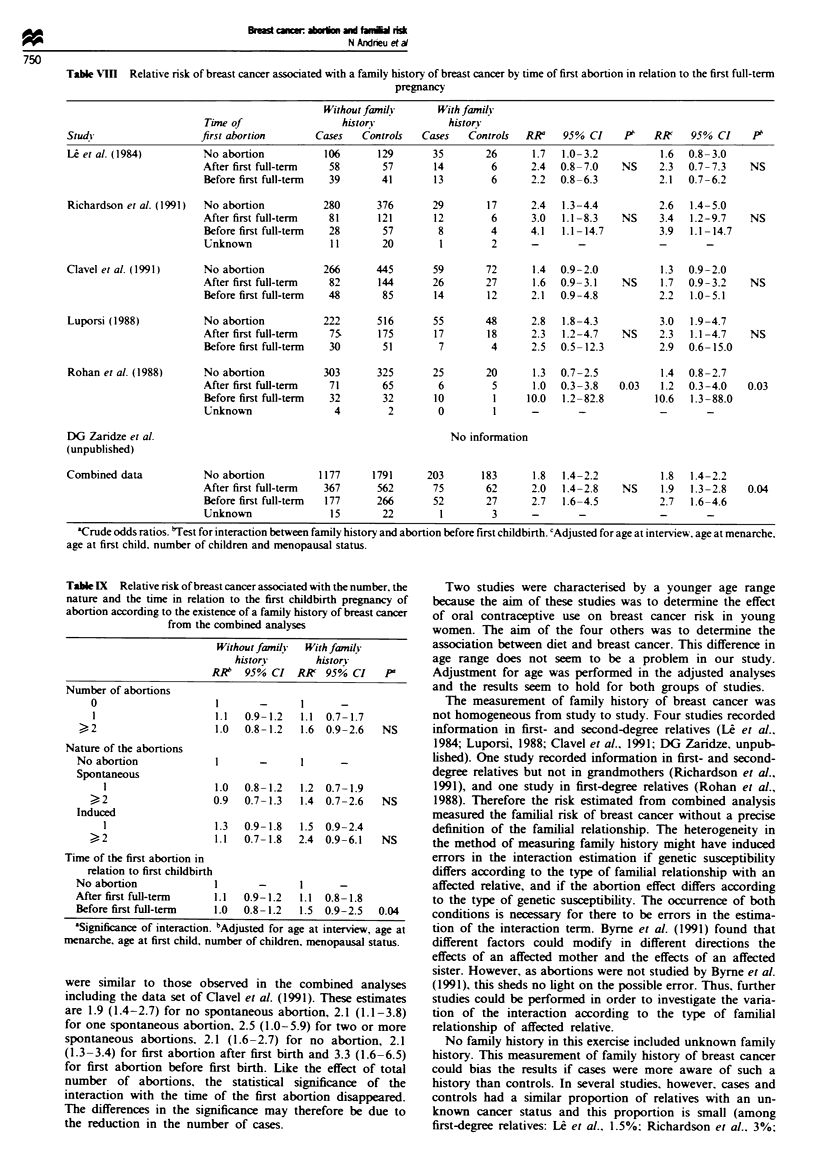

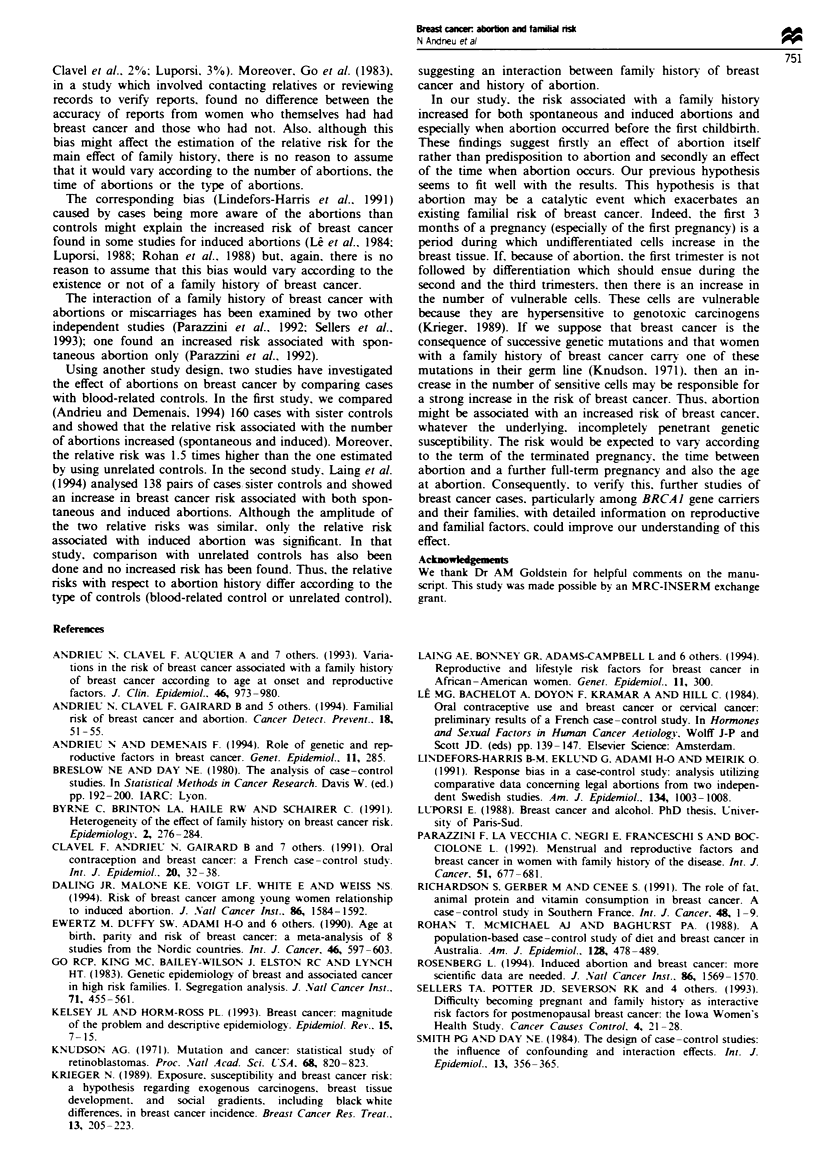


## References

[OCR_01358] Andrieu N., Clavel F., Auquier A., Lê M. G., Gairard B., Piana L., Brémond A., Lansac J., Flamant R., Renaud R. (1993). Variations in the risk of breast cancer associated with a family history of breast cancer according to age at onset and reproductive factors.. J Clin Epidemiol.

[OCR_01362] Andrieu N., Clavel F., Gairard B., Piana L., Brémond A., Lansac J., Flamant R., Renaud R. (1994). Familial risk of breast cancer and abortion.. Cancer Detect Prev.

[OCR_01378] Byrne C., Brinton L. A., Haile R. W., Schairer C. (1991). Heterogeneity of the effect of family history on breast cancer risk.. Epidemiology.

[OCR_01381] Clavel F., Andrieu N., Gairard B., Brémond A., Piana L., Lansac J., Bréart G., Rumeau-Rouquette C., Flamant R., Renaud R. (1991). Oral contraceptives and breast cancer: a French case-control study.. Int J Epidemiol.

[OCR_01388] Daling J. R., Malone K. E., Voigt L. F., White E., Weiss N. S. (1994). Risk of breast cancer among young women: relationship to induced abortion.. J Natl Cancer Inst.

[OCR_01393] Ewertz M., Duffy S. W., Adami H. O., Kvåle G., Lund E., Meirik O., Mellemgaard A., Soini I., Tulinius H. (1990). Age at first birth, parity and risk of breast cancer: a meta-analysis of 8 studies from the Nordic countries.. Int J Cancer.

[OCR_01398] Go R. C., King M. C., Bailey-Wilson J., Elston R. C., Lynch H. T. (1983). Genetic epidemiology of breast cancer and associated cancers in high-risk families. I. Segregation analysis.. J Natl Cancer Inst.

[OCR_01406] Knudson A. G. (1971). Mutation and cancer: statistical study of retinoblastoma.. Proc Natl Acad Sci U S A.

[OCR_01412] Krieger N. (1989). Exposure, susceptibility, and breast cancer risk: a hypothesis regarding exogenous carcinogens, breast tissue development, and social gradients, including black/white differences, in breast cancer incidence.. Breast Cancer Res Treat.

[OCR_01429] Lindefors-Harris B. M., Eklund G., Adami H. O., Meirik O. (1991). Response bias in a case-control study: analysis utilizing comparative data concerning legal abortions from two independent Swedish studies.. Am J Epidemiol.

[OCR_01441] Parazzini F., La Vecchia C., Negri E., Franceschi S., Bocciolone L. (1992). Menstrual and reproductive factors and breast cancer in women with family history of the disease.. Int J Cancer.

[OCR_01449] Rohan T. E., McMichael A. J., Baghurst P. A. (1988). A population-based case-control study of diet and breast cancer in Australia.. Am J Epidemiol.

[OCR_01454] Rosenberg L. (1994). Induced abortion and breast cancer: more scientific data are needed.. J Natl Cancer Inst.

[OCR_01457] Sellers T. A., Potter J. D., Severson R. K., Bostick R. M., Nelson C. L., Kushi L. H., Folsom A. R. (1993). Difficulty becoming pregnant and family history as interactive risk factors for postmenopausal breast cancer: the Iowa Women's Health Study.. Cancer Causes Control.

[OCR_01463] Smith P. G., Day N. E. (1984). The design of case-control studies: the influence of confounding and interaction effects.. Int J Epidemiol.

